# The Impact of Social Support on the Relationship Between Physical Exercise and Cognitive Function in Older Adults: Sociological Perspective

**DOI:** 10.2196/63067

**Published:** 2024-10-04

**Authors:** Yuan Li, Qun Zhai, Weihang Peng

**Affiliations:** 1 Faculty of Health Sciences and Sports, Macao Polytechnic University, Comes Street, Macau Special Administrative Region, 999078, China, 86 68911106, 86 68911106

**Keywords:** social support, physical exercise, cognitive function, elderly, sociological perspective

Our research team recently read the article “Relationship Between Physical Exercise and Cognitive Function Among Older Adults in China: Cross-Sectional Population-Based Study” [1] and was deeply touched. Through rigorous data analysis and detailed research methods, this article reveals the significant relationship between physical exercise and cognitive function in older adults, providing an important theoretical and practical reference for the research field of public health and aging.

Regarding the scientific rigor of this paper in terms of the study design and data analysis, by using data from the China Longitudinal Aging Social Survey, the authors carefully explored the relationship between different exercise intensities and cognitive function through multivariate regression analysis. The results showed that physical activity levels of 500 to 1499 metabolic equivalent of task minutes per week were significantly and positively associated with higher cognitive function scores—a finding that provides an empirical basis for cognitive health intervention strategies in older adults.

However, this paper also has some limitations. The study has a cross-sectional design, which means that a causal relationship between exercise and cognitive function cannot be established. Further, the data mainly rely on self-reports and may be subject to some reporting bias, and these factors may have a significant impact on cognitive function.

From a sociological perspective, future research could delve into the moderating role of social support in the relationship between physical exercise and cognitive function in older adults. Social support includes support from family, friends, the community, and other levels, which can significantly affect the mental health and quality of life of older adults [2]. At present, some studies have explored the impact of social support on the health of older adults from a sociological perspective [1,3]. Good social support can significantly improve the quality of life of older adults and slow down the process of cognitive decline. In addition, participation in community-organized physical activities can effectively improve the cognitive function and mental health of older adults [4,5].

Government and public health agencies should develop policies to encourage and support communities to build age-friendly sports facilities and activity places. At the same time, funding and resources should be provided to support community organizations in organizing regular health activities for older adults. Through family education and publicity, family members’ awareness of the importance of physical exercise for older adults has been improved, and they have been encouraged to actively participate in and support the sports activities of older adults. Communities should actively organize and promote various forms of physical activities for older adults, such as tai chi, square dancing, and vigorous walking, and encourage older adults to participate in volunteer activities to enhance their social ties and mental health.

Overall, we would like to thank the journal for publishing this important research paper that allows us to better understand the effects of physical exercise on cognitive function in older adults from a sociological perspective. We hope to see more such studies in the future to provide more scientific and comprehensive health intervention strategies for the older adult population.
